# Determination of DNA content as quality control in decellularized tissues: challenges and pitfalls

**DOI:** 10.1093/rb/rbae123

**Published:** 2024-10-24

**Authors:** Charlot Philips, Lisanne Terrie, Ewout Muylle, Lieven Thorrez

**Affiliations:** Tissue Engineering Lab, Department of Development and Regeneration, KU Leuven Campus Kulak, 8500 Kortrijk, Belgium; Tissue Engineering Lab, Department of Development and Regeneration, KU Leuven Campus Kulak, 8500 Kortrijk, Belgium; Tissue Engineering Lab, Department of Development and Regeneration, KU Leuven Campus Kulak, 8500 Kortrijk, Belgium; Tissue Engineering Lab, Department of Development and Regeneration, KU Leuven Campus Kulak, 8500 Kortrijk, Belgium

**Keywords:** decellularization, skeletal muscle, DNA quantification, spectrophotometer

## Abstract

Decellularized organs and tissues are emerging within the field of regenerative medicine to meet the growing demand for organ and tissue transplantation. Quality control of these acellular matrices prior to transplantation is of paramount importance to ensure the absence of an adverse reaction. In particular, thorough evaluation of the DNA content is essential but also poses technical challenges. Therefore, in this study, we compared different methods for quantitative and qualitative evaluation of DNA content in native and decellularized skeletal muscle tissue to identify strengths and weaknesses for each. Histological analysis revealed that Feulgen staining is more sensitive and robust than the commonly used hematoxylin–eosin and 4′,6-diamidino-2-phenylindole staining for detection of remaining nuclear material. Furthermore, gel electrophoresis allowed to identify the quality and length of remaining DNA fragments. The results of the quantitative analysis indicated that direct measurement of DNA content in tissue lysates is preferred over silica-based extraction methods, since the latter resulted in the loss of small DNA fragments during extraction. Moreover, a weight loss correction factor should be implemented to take into account the impact of the decellularization on the extracellular matrix. With regard to the detection method, the results revealed that a fluorescence-based approach is more accurate than the use of UV/VIS absorbance. Through combination of the proposed methods, it should be possible to achieve a more standardized evaluation of novel acellular matrices in terms of DNA content and to enhance the predictability of clinical success.

## Graphical Abstract

**Figure:**
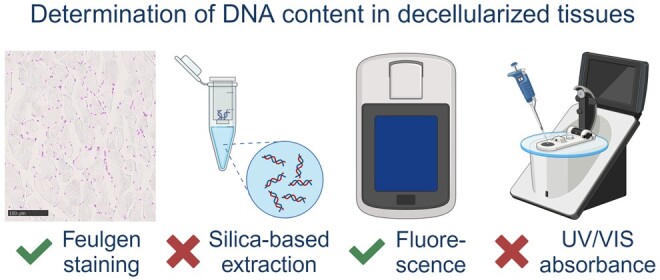

## Introduction

The growing demand for organs and tissues for regenerative medicine is driving the field toward development of novel biomaterials. One interesting approach is to create acellular matrices from human cadaveric donor or even animal sources such as bovine or porcine organs and tissues. These acellular matrices are obtained through a process of decellularization, which removes the cellular content while retaining the extracellular matrix (ECM) and thus, the 3D structure of the organ or tissue [1, 2]. The focus on proper removal of DNA before transplantation stems from the possible negative effect of remaining cellular material on the immune system and eventually the regenerative process [3]. Epitopes on the cell membrane or damage-associated molecular pattern molecules could elicit an adverse immune response, potentially leading to graft rejection.

At the moment, several commercial acellular matrices are available, including Tutopatch^®^ (bovine pericard), AlloDerm^®^ (human dermis), Restore™ (porcine small intestinal submucosa) and MatriStem^®^ (porcine urinary bladder matrix) [1, 4]. These ECM-based scaffolds are thin, lamellar structures and provide limited mechanical support. Commercial products derived from more complex tissues or entire organs are still lacking. For thicker tissues and organs, more advanced and potentially harsher decellularization methods are required to remove all DNA content, resulting in more damage to the ECM. Additionally, it is also more challenging to accurately determine the total DNA content in these scaffolds. Since the majority of the DNA content is highly fragmented, it is not as straightforward to use conventional detection techniques.

Several methods to qualitatively and quantitatively evaluate DNA in biological samples exist. Quality control of acellular matrices can be performed through histological assessment, of which staining with hematoxylin–eosin (H&E) and 4′,6-diamidino-2-phenylindole (DAPI) are most commonly used. For quantitative analysis, samples are first processed to solubilize the DNA content, followed by either fluorescence-based or UV/VIS absorbance-based detection. Processing methods prior to quantification include tissue digestion, followed by solid phase silica-based extraction, organic extraction or salting out [5]. This variety of available methods also results in a variety of outcome values for DNA content in native and decellularized tissues. For example, reported values of DNA content in native skeletal muscle tissue range from ∼100 ng/mg dry weight [6–8] up to 2920 ng/mg dry weight [9].

The goal of this study is to evaluate different methods to determine DNA content in decellularized skeletal muscle tissue and to identify strengths and weaknesses for each of these methods. In this way, a more informed selection can be made for future quality control of acellular matrices, which will enhance standardization within the field.

## Materials and methods

### Decellularization of skeletal muscle tissue

Porcine skeletal muscle tissue was harvested from the biceps femoris of freshly euthanized pigs (13–16 weeks old; Orsi Academy). During transportation, the tissue was kept in high glucose Dulbecco’s Modified Eagle Medium (Gibco; 31966021), after which it was cut into beam-shaped samples of 5 × 5 × 20 mm and stored at −80 °C until the start of the decellularization. Untreated samples served as native control tissue, while four different decellularization protocols were applied as previously described [10–13]. All steps were performed under constant agitation and at room temperature unless stated otherwise.

The first protocol was developed in-house, based on the method described by Roosens *et al.* [14], Philips *et al.* [15] and Terrie *et al*. [13]. The decellularization started with overnight incubation in a 50 mM Tris buffer (pH 8) at 4 °C, followed by 1% Triton X-100 (Sigma-Aldrich; 93443) for 24 h at 4 °C. Subsequently, samples were washed twice with Hank’s Balanced Salt Solution (HBSS; Sigma-Aldrich; H1387), followed by an enzymatic treatment with 40 000 Kunitz units/l DNase I (Goldbio; D-300-1) and 100 mg/l trypsin (Sigma-Aldrich; T9201) in HBSS for 2 × 3 h at 37 °C. Samples were then again placed in 1% Triton X-100 for 18 h at 4 °C. A final wash with HBSS at 4 °C for 4 days was performed, after which the samples were stored in fresh HBSS at 4 °C until further use.

For the second protocol, based on Wolf *et al*. [10], samples were first lyophilized (Analis; Alpha 1-4 LDplus) and then incubated for 2 h in 2:1 (v/v) solution of chloroform/methanol at 4 °C to extract the lipids. Next, rehydration was performed through a degrading series of ethanol (100%, 90%, 70%, 50% and 0%) for 30 min each at 4 °C. Subsequently, an enzymatic treatment with 0.2% Trypsin/0.2% EDTA for 2 h at 37 °C followed to remove cellular material. After that, a series of chemical treatments with 2% sodium deoxycholate (SDC; Sigma-Aldrich; D6750) for 5 h, fresh 2% SDC for 16 h, 1% Triton-X 100 for 1 h, and finally 0.1% (w/v) peracetic acid/4% (v/v) ethanol, all at 4 °C, was performed. Samples were washed once with distilled water and twice with PBS for 30 min each at 4 °C between each incubation step. Finally, samples were stored in fresh PBS at 4 °C until further use.

For the third protocol, based on Porzionato *et al*. [11], samples were first washed with distilled water for 24 h at 4 °C, after which they were incubated for 1 h with 0.05% trypsin/0.02% EDTA (Gibco; 25300054) at 37 °C. Next, samples were briefly washed in PBS and further incubated with 2% Triton X-100—0.8% NH_4_OH (Sigma-Aldrich; 318612) for 72 h at 4 °C. Finally, samples were incubated in distilled water for 48 h at 4 °C followed by three washes in PBS. Samples were stored in fresh PBS at 4 °C until further use.

The fourth protocol was based on De Coppi *et al*. [12] and started with four rinses with PBS. Subsequently, the samples were incubated in distilled water for 72 h at 4 °C, followed by 4% SDC for 4 h at 4 °C and 2000 Kunitz units/l DNAse I in 1M NaCl for 3 h at 37 °C. These incubation steps were repeated once after which the samples were stored in PBS at 4 °C until further use.

### Histological analysis

Histological samples were fixed in 4% paraformaldehyde for 24 h at 4 °C. After fixation, samples were dehydrated with increasing alcohol series (70%, 80%, 90% and 100%) and embedded in paraffin. To make sure the inner part of the samples could be visualized, samples were cut in half and positioned with the inner part toward the bottom of the mold. Finally, paraffin blocks were sectioned into 5 µm thick sections with a microtome (Leica RM2125 RTS). DNA content was visualized with a H&E staining as well as a Feulgen staining. In addition, a fluorescent staining with DAPI (Invitrogen; P36935) was performed.

### DNA quantification

To quantify the DNA content, samples were first lyophilized and dry weight of each 20 × 5 × 5 mm sample was noted down to allow the calculation of a weight loss correction factor. This correction factor was defined as follows:

$$\mathrm{correction}\;\mathrm{factor}=1-\frac{\mathrm{weight}\;\mathrm{native}\;\mathrm{tissue}\;\left(\mathrm{mg}\right)-\mathrm{weight}\;\mathrm{decellularized}\;\mathrm{tissue}\;\left(\mathrm{mg}\right)}{\mathrm{weight}\;\mathrm{native}\;\mathrm{tissue}\;\left(\mathrm{mg}\right)}.$$

**Figure 1.**
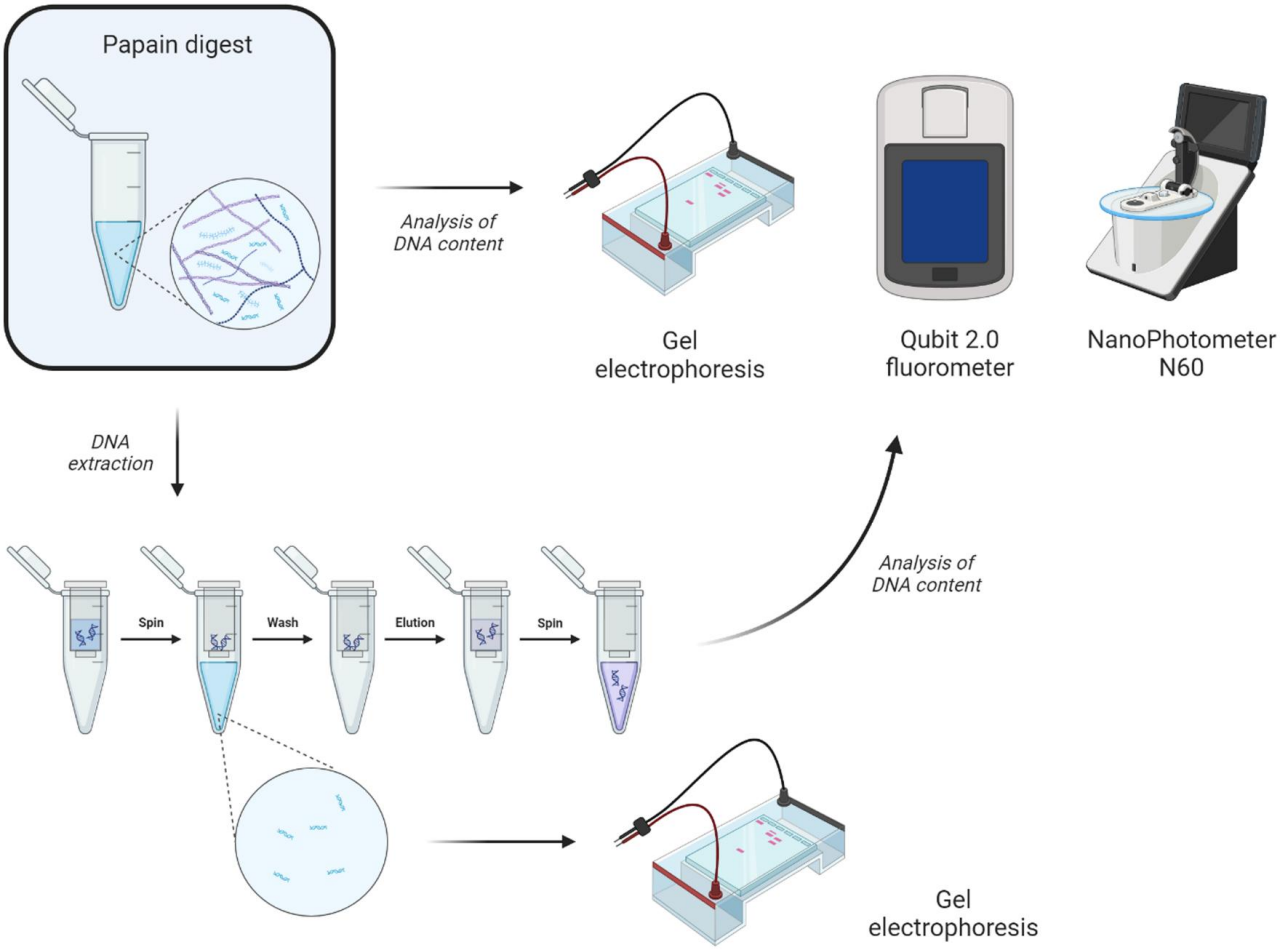
Schematic representation of evaluation methods for DNA content. Native and decellularized tissue samples are first digested with papain, followed by immediate analysis of DNA content or further extraction of DNA with the DNeasy blood and tissue kit. The first flow-through (usually discarded) during extraction is also used for gel electrophoresis.

Next, the lyophilized samples were enzymatically digested, as previously described [13], by adding 1 ml papain solution to ∼10 mg of dry tissue. The papain solution was composed of a 0.2 M sodium phosphate buffer (pH 6.4) containing 8 mg/ml sodium acetate, 4 mg/ml EDTA disodium salt, 0.8 mg/ml cysteine-HCl and 5 µl/ml papain (Sigma-Aldrich; P3125). The samples were incubated for 18 h at 65 °C, followed by centrifugation for 10 min at 10 000 g to precipitate any remaining debris. The supernatant was further used for quantitative analysis with the Qubit dsDNA BR Assay Kit (Thermo Fisher Scientific; Q32850) and qualitative analysis with gel electrophoresis, or further purified with the DNeasy Blood and Tissue Kit (Qiagen; cat. no. 69504) according to the manufacturer’s recommendations (Figure 1). After purification, samples were again analyzed with the Qubit dsDNA BR Assay Kit and a Qubit 2.0 Fluorometer as well as with a NanoPhotometer^®^ N60 (Implen, Germany).

### Gel electrophoresis

To visualize the length of DNA fragments at different stages of the DNA quantification, gel electrophoresis was performed on three points in the process (Figure 1). A first gel was run immediately after papain digestion. The second gel contained the first flow-through of the silica-based extraction, while the third gel was performed at the end of the silica-based extraction. Each sample of 20 µl was mixed with 4 µl of 6X TriTrack DNA Loading Dye, as previously described [13]. Next, 20 µl of this mixture was loaded on a 1% agarose gel with GelRed^®^ nuclear stain (VWR; 41011) to separate the DNA fragments. As a reference, both a 1 kb and 100 bp DNA ladder (Thermo Fisher Scientific; SM0311 and SM0242) were included. Visualization was performed with an E-Gel^®^ Imager (Invitrogen) using the GelCapture EGel 2.2.4 software.

### Statistical analysis

Quantitative measurements of DNA content were statistically analyzed with GraphPad Prism 8.0.1. Multiple *t*-tests corrected with the Holm–Sidak method were performed to evaluate differences between the different DNA quantification methods. *P*-values <0.05 were considered statistically significant.

## Results

### Histological analysis

**Figure 2.**
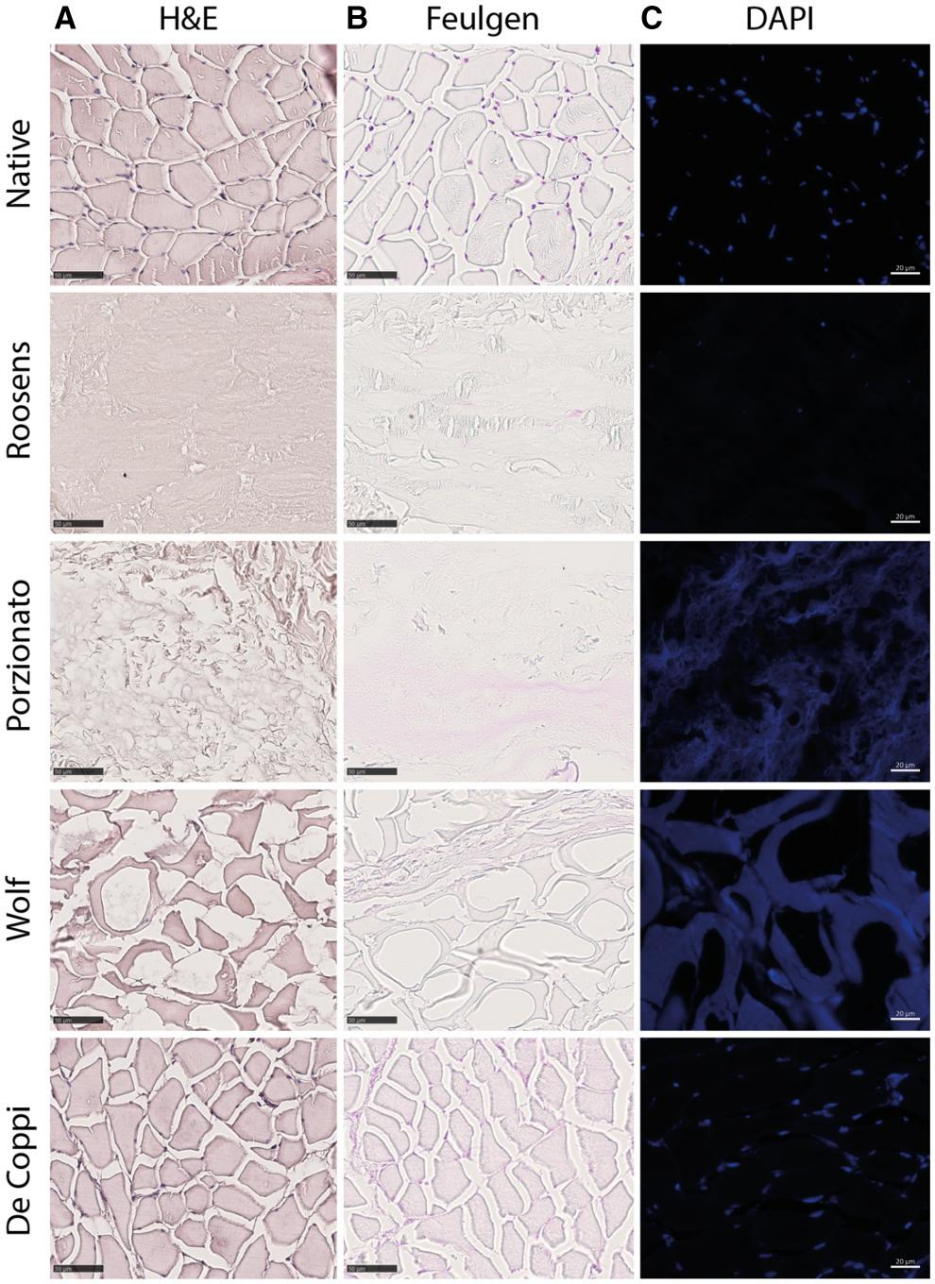
Histological evaluation of native and decellularized skeletal muscle tissue. (**A**) Hematoxylin–eosin staining demonstrates presence of nuclei in the native and De Coppi group. No clear nuclei are observed in the Roosens, Porzionato and Wolf group. Scale bar = 50 µm. (**B**) Feulgen staining shows intact nuclei in the native and De Coppi group (pink dots), while DNA fragments are found in the Porzionato and Wolf group (pink smear). No nuclear material is visible in the Roosens group. Scale bar = 50 µm. (**C**) DAPI staining demonstrates presence of nuclei in the native and De Coppi group, while in the Roosens group no nuclei can be seen. For the Porzionato and Wolf group, a uniform blue staining is visible, indicating autofluorescence of the samples. Scale bar = 20 µm.

The impact of the different decellularization methods on the DNA content was first analyzed through histological staining with H&E, Feulgen and DAPI (Figure 2). Native tissue is characterized by the presence of clear nuclei, visualized as purple dots with H&E, pink dots with Feulgen and blue dots with DAPI staining. Samples from the De Coppi group are clearly not decellularized, as intact nuclei can still be detected with each of the staining methods. For the other three groups, no apparent nuclei can be seen with H&E staining. This absence of nuclear material is confirmed with Feulgen and DAPI staining for samples of the Roosens group. However, a pink smear is visible with Feulgen staining for samples of the Porzionato group and to a lesser extent the Wolf group, indicating the presence of DNA fragments. Interestingly, the pink color is mainly localized within the connective tissue. On the DAPI staining, the DNA fragments are more difficult to distinguish, since a uniform blue staining is observed, pointing toward autofluorescence of the samples.

### DNA quantification

To quantitatively determine the DNA content, different methods were compared to identify the most appropriate strategy. Measurements were either performed immediately after digestion with papain or after further extraction with silica-based columns. In addition, quantification based on fluorescence (Qubit) was compared to UV/VIS absorbance (NanoPhotometer). Accurate measurement of the DNA content after papain digestion with the NanoPhotometer, however, was not feasible due to the impurity of the samples. The high protein content in these samples resulted in a 260/280 absorbance ratio far from the required values of 1.8–2 (data not shown).

Based on the dry weight of the native and decellularized tissues, a weight loss correction factor was first calculated (Table 1). The significant weight loss (*P* < 0.01) for samples of the Porzionato group compared to the native group resulted in a correction factor of 0.06, while the weight loss between the Roosens and Wolf group was similar, resulting in correction factors of 0.23 and 0.28, respectively. Samples of the De Coppi group did not show a significant weight loss compared to the native group, resulting in a correction factor of 0.64.

**Table 1.** DNA content based on fluorescence after papain digestion, with and without the weight loss correction factor

	Measured DNA content (ng/mg)	Tissue weight (mg)	Correction factor	Corrected DNA content (ng/mg)
Native	993.0 ± 76.9	164.8 ± 36.8	1.00	993.0 ± 76.9
Roosens	167.0 ± 49.6	37.9 ± 7.1	0.23	38.4 ± 11.4
Porzionato	1815.8 ± 353.9	10.6 ± 2.0	0.06	116.4 ± 22.7
Wolf	291.0 ± 45.3	46.3 ± 5.5	0.28	81.7 ± 12.7
De Coppi	461.9 ± 25.5	105.7 ± 19.1	0.64	296.2 ± 16.3

**Figure 3.**
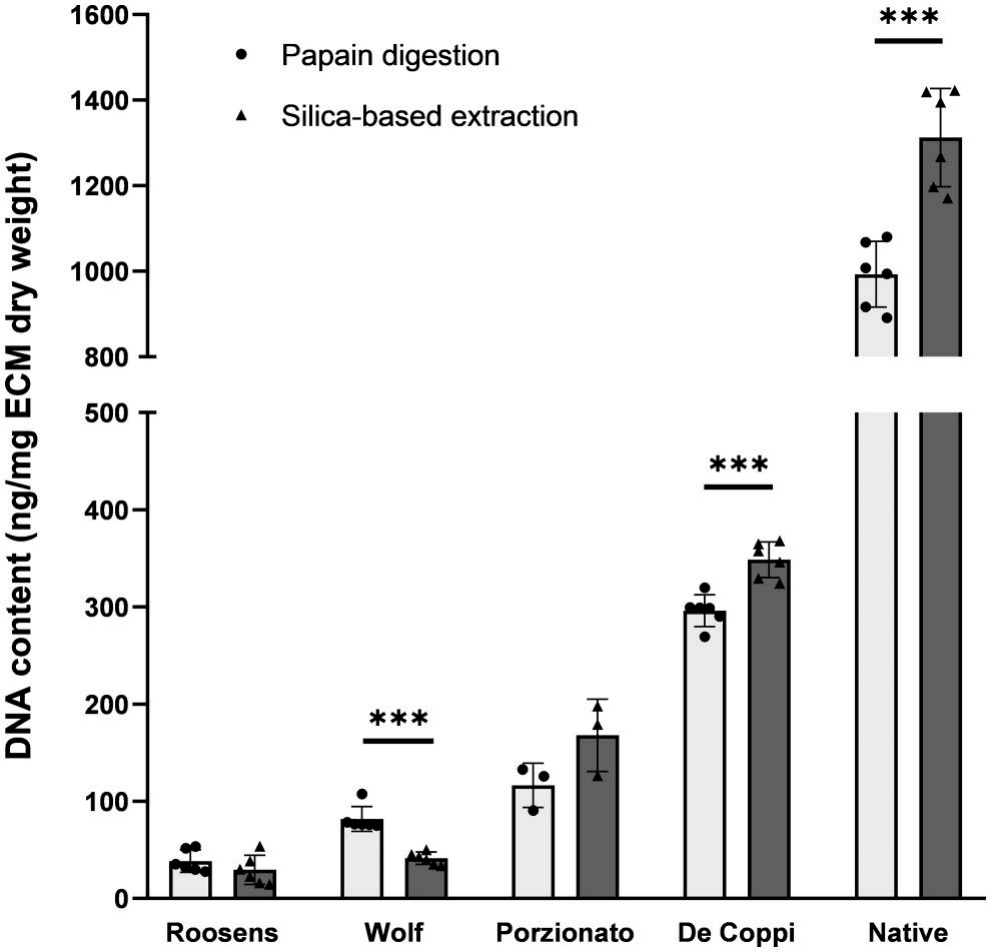
Determination of DNA content based on fluorescence after papain digestion or silica-based extraction. For samples with a high decellularization efficiency (Roosens and Wolf), lower values for DNA content are found after silica-based extraction compared to papain digestion alone. For native samples as well as samples with a low decellularization efficiency (Porzionato and De Coppi), the opposite is observed. ***P < 0.001 (*n* = 6, except for Porzionato where *n* = 3).

The corrected DNA content based on fluorescence is shown in Figure 3, comparing the measurements after papain digestion and silica-based extraction. For samples of the Roosens and Wolf group, which have a strong reduction of DNA content after decellularization, lower values were detected after silica-based extraction compared to papain digestion. This was even significant for samples of the Wolf group (*P* < 0.001). On the other hand, for samples of the Porzionato and De Coppi group, which were not efficiently decellularized, as well as for the native group, higher values were detected after silica-based extraction compared to papain digestion. This increase was significant for the De Coppi and native group (*P* < 0.001).

**Figure 4.**
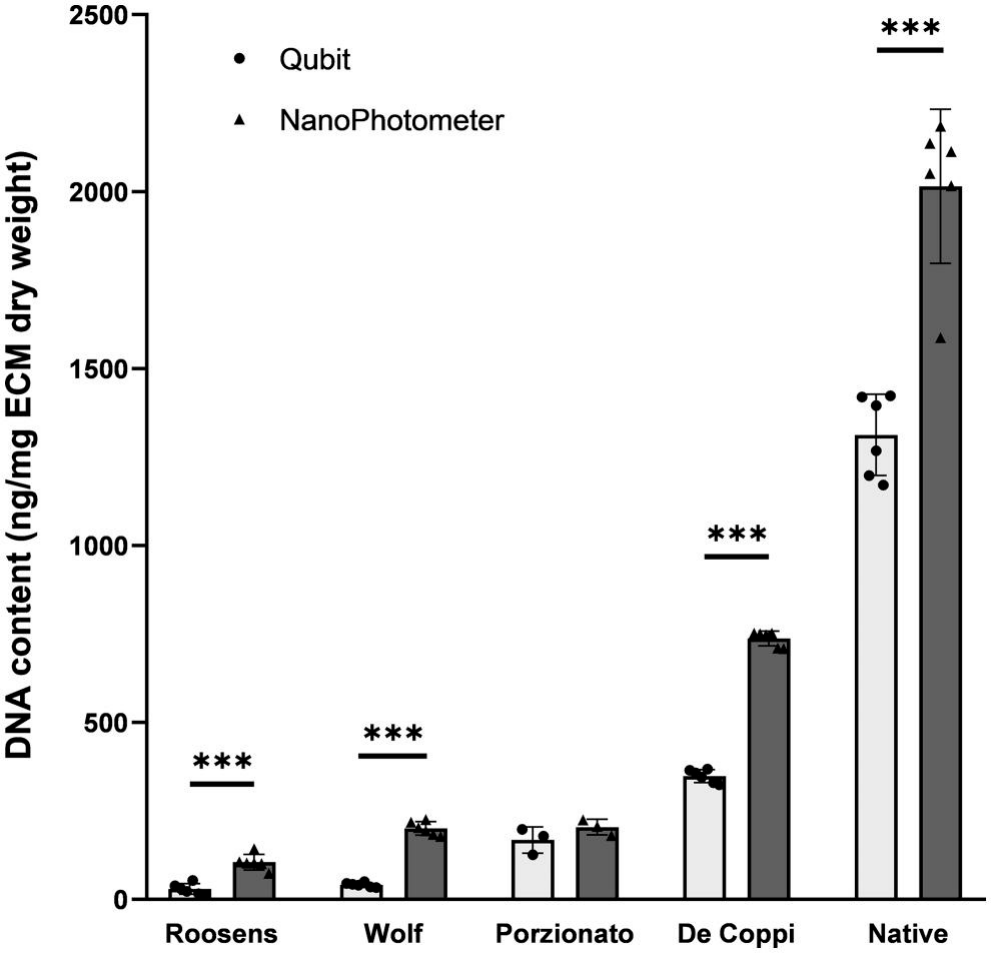
Determination of DNA content with Qubit (fluorescence) or NanoPhotometer (UV/VIS absorbance) after silica-based extraction. Higher DNA values are observed for all groups after measurement with a NanoPhotometer compared to Qubit. These differences are significant, except for the Porzionato group. ***P < 0.001 (*n* = 6, except for Porzionato where *n* = 3).

In Figure 4, the difference between fluorescence-based and UV/VIS absorbance-based measurements after extraction with silica-based columns is depicted. For all sample groups, higher values for DNA content are observed when measuring with the NanoPhotometer (UV/VIS absorbance) compared to Qubit (fluorescence). For each of the groups, this difference was significant (*P* < 0.001), except for the Porzionato group. Moreover, it seems that the difference between both methods is more pronounced with increasing decellularization efficiency and thus a lower total DNA content. A 3.56 and 4.85 fold change is observed for samples of the Roosens and Wolf group, respectively, while this fold change is only 2.12, 1.54 and 1.22 for the De Coppi, native and Porzionato groups, respectively.

### Gel electrophoresis

**Figure 5.**
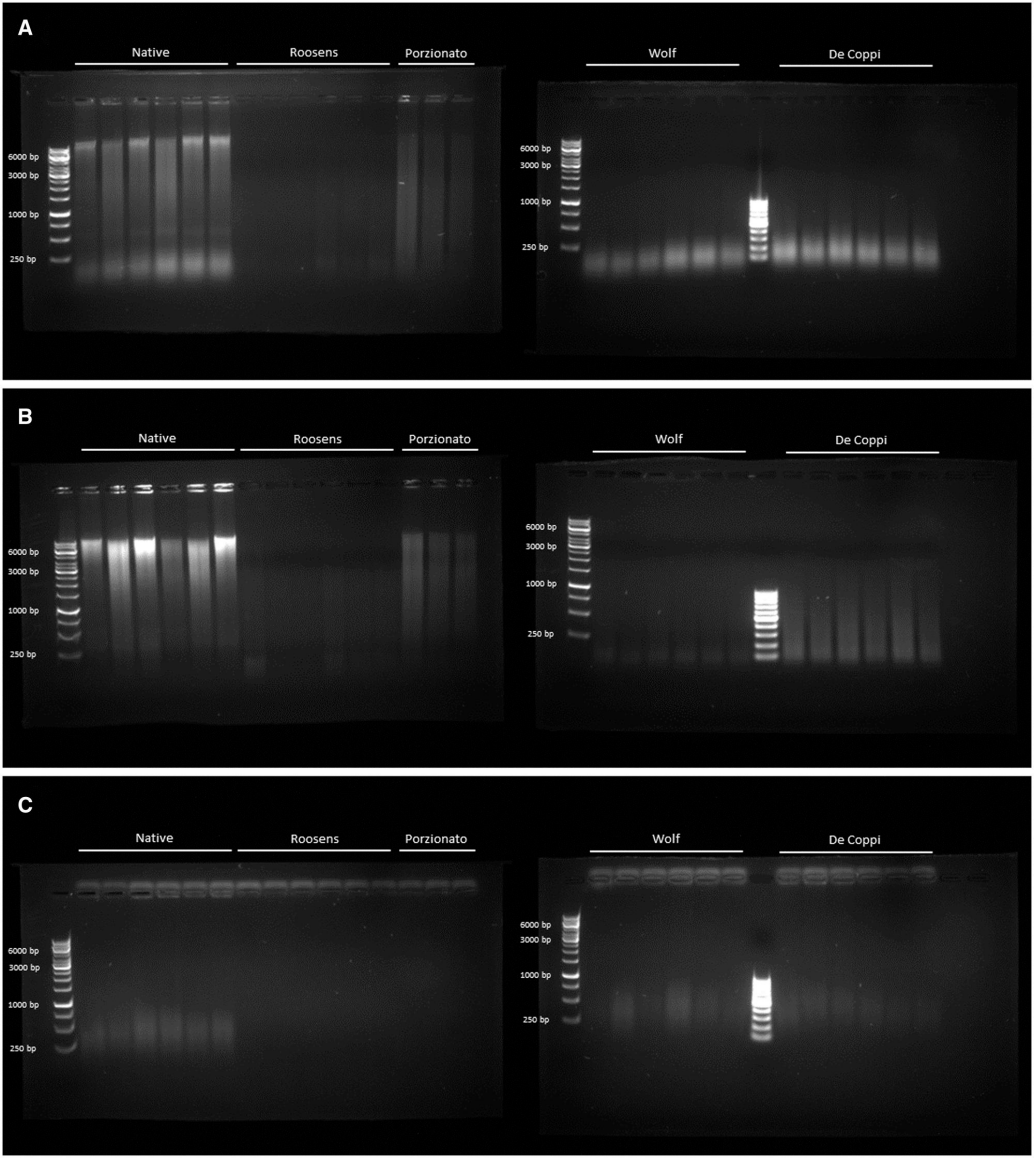
Gel electrophoresis of DNA isolated from native and decellularized skeletal muscle tissue. (**A)** DNA obtained immediately after papain digestion. (**B**) DNA obtained after silica-based extraction. (**C**) DNA present in the first discard during silica-based extraction. A 1 kb and 100 bp ladder are included as references. *n* = 6 except for Porzionato where *n* = 3.

The DNA content, and more specifically quality and length of the DNA fragments, was further analyzed with gel electrophoresis (Figure 5). When running the gel immediately after papain digestion, it was found that samples of the native group consisted of mainly large DNA fragments of >10 000 bp, with presence of smaller fragments of <250 bp as well (Figure 5A). Samples from the Porzionato group also contained DNA fragments of different sizes, ranging from 250 to 10 000 bp in length. However, these fragments were less abundant compared to native samples and no fragments smaller than 250 bp were observed. In contrast, the DNA content of samples of the Wolf and De Coppi group almost exclusively contained DNA fragments smaller than 250 bp. For samples of the Roosens group, no clear DNA fragments could be observed.

Samples of the native group extracted with the silica-based columns did not contain any DNA fragments smaller than 250 bp (Figure 5B). Larger fragments, and in particular those having a length of >10 000 bp were present in large quantities. Samples of the Porzionato group consisted of fragments between 1000 and 10 000 bp. On the other hand, DNA fragments of samples of the De Coppi group ranged from 100 to 1000 bp. For the Wolf group, DNA fragments were again limited to sizes smaller than 250 bp. Interestingly, both for the De Coppi and Wolf group, fragments smaller than 250 bp seemed to be less abundant (lower intensity) compared to samples of the same groups but only digested with papain and not extracted with silica-based columns. For samples of the Roosens group again no apparent DNA fragments were observed.

A third gel was run to visualize the presence of DNA fragments in the first discard (flow-through) during silica-based extraction (Figure 5C). For samples of the Roosens and Porzionato group, no DNA fragments were detected. For samples of the native, Wolf and De Coppi group, on the other hand, fragments between 250 and 500 bp were visible.

## Discussion

Determination of total DNA content is an important aspect in the evaluation of acellular matrices to assess their clinical applicability. In this context, it is crucial to avoid an adverse immune response upon transplantation. Since different methods for visualization and quantification of DNA are available, it is unclear which methodology will most accurately represent the total DNA content. Therefore, in this study, we compared different quantitative and qualitative methods to identify strengths and weaknesses for each.

Although several acellular matrices are already commercially available, the U.S. Food and Drug Administration has no clear regulations when it comes to DNA content [16]. Most researchers are adhering to the minimal criteria defined by Crapo *et al*. [1], stating that acellular matrices should have (i) <50 ng dsDNA per mg ECM dry weight, (ii) <200 bp DNA fragment length and (iii) lack of visible nuclear material in tissue sections stained with DAPI or H&E. However, based on our histological analysis of different decellularized skeletal muscle samples, it seems that H&E staining is not sensitive enough to identify nuclear remnants. Only intact nuclei can be detected, while decellularized samples often contain DNA fragments, which are not picked up by this method. On the other hand, DAPI staining allows to detect small DNA fragments but has the downside of being fluorescence-based. Strong autofluorescent signals, as we observed for the Porzionato and Wolf group, can mask the DAPI signal originating from binding with DNA. It is not surprising that decellularized samples can exhibit autofluorescence, as they are mainly composed of collagen, which is known to possess high quantum yields leading to a strong fluorescent signal upon excitation [17]. In addition, detergents such as Triton X-100 used for decellularization can also result in autofluorescence if they are not properly washed away from the samples [18]. To counteract the disadvantages of both H&E and DAPI staining, we propose to implement the Feulgen staining as a more sensitive and robust detection method of DNA content. The Schiff’s reagent used in the staining reacts with DNA molecules with the development of a pink color proportional to the DNA concentration. Moreover, spatial information is also provided due to the faint background staining of the tissue.

To evaluate the first criterion of Crapo *et al*., having <50 ng dsDNA per mg ECM dry weight, several quantitative methods are available. The most commonly used method is solid-phase silica-based extraction with commercial kits such as the DNeasy Blood and Tissue Kit of Qiagen or the PureLink™ Genomic DNA Mini Kit of Invitrogen [5]. However, recent reports are showing that the use of silica-based columns results in a loss of small DNA fragments (<200 bp) and thus an underestimation of total DNA content [19, 20]. This was also observed in the present study, with a clear loss of small DNA fragments in the native, Wolf and De Coppi group as visualized with gel electrophoresis. Samples from the Roosens and Porzionato group did not contain detectable amounts of small DNA fragments before silica-based extraction, and thus, no apparent signal could be seen in the first flow-through either. These results confirm that the use of silica-based columns are not the most optimal approach when determining DNA content from decellularized tissues. Silica-based columns are designed to purify genomic DNA and rely on electrostatic and hydrophobic interaction of the negatively charged DNA with the positively charged silica [21]. The smaller the DNA fragments, the weaker the interaction with the silica and the higher the chances of losing the DNA fragments during the extraction. Therefore, direct measurement of DNA content from the tissue lysate seems a more appropriate approach to determine total DNA content in decellularized tissues.

Surprisingly, when quantifying DNA content with the Qubit dsDNA BR Assay before and after silica-based extraction, two opposing trends are observed. On the one hand, silica-based extraction resulted in lower values of DNA content for samples decellularized with the most efficient protocols, i.e. the Roosens and Wolf protocol. This is probably a consequence from the loss of small DNA fragments as shown with gel electrophoresis upon extraction. In contrast, for samples still containing large DNA fragments and intact nuclei, i.e. the native, Porzionato and De Coppi group, higher values of DNA content were observed after silica-based extraction. This counterintuitive observation might be explained by differences in confounding factors before and after extraction. It has been shown that the accuracy of fluorescent dyes such as PicoGreen, similar to the Qubit used in the present study, is affected by fragmentation of DNA [22–24]. More specifically, lower concentrations will be measured with higher degrees of DNA fragmentation. In addition, PicoGreen will also bind to RNA and ssDNA which produces only 10% of the fluorescent signal compared to dsDNA [25]. After extraction, smaller DNA fragments (<200 bp) and impurities such as RNA and ssDNA are removed, resulting in more dye available for binding to large dsDNA and hence leading to a stronger fluorescent signal.

Since a vast amount of decellularization studies employs UV/VIS absorbance rather than fluorescence for determination of DNA content [7, 8, 11, 26–30], we also compared both methods in the present study. Consistently higher values were found with the NanoPhotometer in comparison to the Qubit dsDNA BR Assay after silica-based extraction, as has been repeatedly reported by other research groups [22, 23, 31]. These higher values can most likely be attributed to the presence of residual proteins, since the NanoPhotometer will detect any signal at 260 nm wavelength regardless if it originates from DNA or protein. This non-specificity also renders UV/VIS absorbance-based detection methods unsuitable for direct measurement of DNA content in tissue lysates, as they contain high amounts of protein as well as RNA and ssDNA.

In addition to selecting the best methodology for processing and measuring DNA content in acellular matrices, another challenge is how to express the DNA content. It is generally accepted to express DNA content as ng per mg ECM dry weight. However, this assumes that the decellularization protocol will have little effect on the ECM composition. Since more dense tissues such as skeletal muscle will require more extensive decellularization, it is inevitable to have some impact on the ECM as well. For example, the Porzionato protocol described in the present study had a drastic impact on the ECM of the samples (data not shown) but less impact on the DNA content. This eventually resulted in a ratio higher than native samples. Therefore, we introduced a weight loss correction factor to take into account weight differences compared to the native samples. The impact this correction factor can have, emphasizes that caution should be taken when interpreting quantitative values for DNA content. Indeed, some commercially available acellular matrices are reported to have a DNA content up to 2 µg/mg ECM dry weight [32]. These values greatly exceed the threshold put forward by Crapo *et al.*, but might not take into account the impact on the ECM since no correction factor is mentioned. The positive outcomes associated with these commercial matrices further highlight the delicate balance between removal of all cellular content and preservation of the ECM in terms of structure and composition when developing a decellularization protocol. Adequate retention of the ECM components is required for a favorable immune response upon implantation, by shifting macrophage polarization from a proinflammatory to a proremodeling phenotype [33].

Finally, our observations also underscore the importance of correlating quantitative methods with qualitative methods such as histological analysis and gel electrophoresis. The histological images are in agreement with the DNA quantification, revealing the Roosens protocol as the most efficient and the De Coppi protocol as the least efficient for skeletal muscle tissue decellularization. By applying the weight correction factor, the quantitative differences are even more pronounced and correlate even better with the histological observations.

## Conclusion

Based on our results, it is advisable to use both qualitative and quantitative methods for quality control of acellular matrices. Histological analysis with a Feulgen staining could give valuable information on the presence and distribution of remaining nuclear material, while gel electrophoresis allows to identify the length of DNA fragments. Furthermore, for quantitative analysis, direct measurement of DNA content in tissue lysates is preferred over silica-based extraction methods and fluorescence-based detection seems more appropriate than UV/VIS absorbance-based detection. Moreover, a weight loss correction factor should be applied to take into account the impact on the ECM. Through combination of the proposed methods, it should be possible to achieve a more standardized evaluation of novel acellular matrices in terms of DNA content and to enhance the predictability of clinical success.
